# The relationship between natural rain intensity and Ascochyta blight in chickpea development

**DOI:** 10.1007/s10658-022-02538-2

**Published:** 2022-09-24

**Authors:** Ihsanul Khaliq, Kevin Moore, Adam H. Sparks

**Affiliations:** 1 University of Southern Queensland, Centre for Crop Health, Toowoomba, Queensland 4350 Australia; 2 New South Wales Department of Primary Industries, 4 Marsden Park Rd, Tamworth, NSW 2340 Australia; 3 Department of Primary Industries and Regional Development (DPIRD), Perth, WA 6000 Australia

**Keywords:** Splash dispersal, Spore dispersal, Quantitative epidemiology, *Ascochyta rabiei*, Rain splash, Conidial dispersal

## Abstract

Ascochyta blight management strategy in chickpea standing crops in Australia is solely based on applying protective fungicides before a forecast rainfall event. Despite this, studies on the likely interaction between natural rain (as well as simulated rain) amount, duration and Ascochyta blight development are rare. This study was conducted to investigate the relationship between natural rain intensity (mm/h) and Ascochyta blight development. Infested chickpea residue were placed at the soil surface, and three pots of a susceptible chickpea cultivar were randomly placed on each side of the plot (total 12 pots and 36 plants), preceding a forecast rainfall event. Trap plants were transferred to a controlled temperature room after rain events. After a 48 h incubation period, trap plants were transferred to a glasshouse to allow lesion development. The number of lesions on all plant parts were counted after two weeks. Lesions developed in rain amounts as low as 1.4 mm and rain durations as short as 0.7 h. The number of lesions significantly increased with increasing rain amount. There was a positive effect of increasing rain duration and a negative effect of increasing wind speed. This study suggests that small rain amounts, shorter duration rains or a limited amount of primary inoculum are not barriers to conidial dispersal or host infection, and that the current value of a rainfallthreshold (2 mm) for conidial spread and host infection is not accurate for susceptible cultivars.

## Introduction

Chickpea (*Cicer arietinum*) is the second most important grain legume crop after soybean globally with a production value of more than US$7.6 B between 2014 and 2016 (FAOSTAT, 2019). Ascochyta blight, caused by *Ascochyta rabiei* (syn. *Phoma rabiei*), is the most important constraint to chickpea production worldwide (Gayacharan et al., 2020; Nene, 1982; Pande et al., 2005). Yield losses from Ascochyta blight can be up to 100% in susceptible cultivars under favourable conditions for the disease (Nene, 1982). *Ascochyta rabiei* is known to survive on infested residue, infected seed and volunteer chickpea plants (Pande et al., 2005). The survival period on infested residue is up to 2 years when infested residues are at the soil surface, but viability is lost within 5 months when infested residues are 5–40 cm deep (Kaiser, 1973). Infested residue and volunteer chickpea plants serve as the main source of inoculum for disease initiation in Australia as very high levels of control of seed-borne infection (approaching 100%) can be achieved via commercial seed treatments. Infected seeds are not considered important in Ascochyta blight epidemiology unless growers are sowing untreated or poorly treated seed (Kevin Moore’s unpublished data). *Ascochyta rabiei* is heterothallic, however only one mating type has been found in Australia (Leo et al., 2016; Mehmood et al., 2017). The sexual stage/spores of the fungus are absent in Australia, and thus conidia are the only spore type involved in the disease cycle (Coventry, 2012). Infection and disease development are favoured by temperatures between 10 and 30 °C with an optimum at 20 °C (Kaiser, 1973), relative humidity >95% (Nene, 1982), and leaf wetness period of at least 6–10 h (Moore et al., 2016). Moderate resistance to Ascochyta blight can be overcome when chickpea plants are exposed to intermediate to high disease pressure under cool (20 °C) and wet (> 90% humidity) conditions (Chongo et al., 2003; Coventry, 2012). To date, there is no resistant variety available against Ascochyta blight in Australia. All varieties are susceptible to moderately susceptible (https://www.nvtonline.com.au/).

Rain is required to disperse *A. rabiei* conidia, heavy dew or high relative humidity alone will not cause significant disease spread (Moore et al., 2016). Briefly, pycnidia absorbe moisture during rain and conidia within pycnidia are exuded through the ostiole in a cirrhus by hydrostatic pressure. When rain drops strikes a cirrhus, the kinetic energy breaks up the conidial mass and disperse conidia to nearby plants, thus spreading the disease (Coventry, 2012). The amount of kinetic energy varies with the size of rain drops, rain duration and intensity (Sache, 2000). Smaller rain drops have less kinetic energy, and thus remove fewer spores (Sache, 2000). *Ascochyta rabiei* conidia are primarily dispersed by rain splash up to 1 m (Kimber, 2002), but a recent study showed that conidia travel at least 75 m in wind-driven rain (Khaliq et al., 2020). Conidia can also be washed off from upper leaves to lower leaves, or can be scrubbed from aerosols (liquid suspension of conidia in air) and deposited on chickpea plants when falling rain drops pass through the aerosol (Coventry, 2012).

There are significant gaps in the knowledge about the epidemiology of Ascochyta blight due to the historic emphasis on developing resistant cultivars and fungicides for the disease management (Pande et al., 2005). Rain is an environmental variable with many intrinsic characteristics, such as amount and duration (Pruppacher & Klett, 1978). Spore dispersal in splash dispersed pathogens is linked to the intrinsic characteristics of rain (Madden et al., 1996). Studies on the relationship between the intrinsic characteristics of natural rain and Ascochyta blight development are rare; however, studies conducted on other plant pathogens can provide useful insights into understanding the relationship between the intrinsic characteristics of natural rain and Ascochyta blight development. A positive relationship between increasing rain amount and duration and conidial dispersal has been established for a splash dispersed pathogen, *Colletotrichum acutatum* (Ntahimpera et al., 1997). Therefore, a positive relationship between increased rain duration and rain amount and *A. rabiei* conidial dispersal is plausible. As *A. rabiei* conidia are mainly dispersed by rain splash, a minimal or no association between conidial dispersal and wind speed is also plausible.

In Australia, chickpea growers’ Ascochyta blight management strategy in a standing crop is solely based on applying fungicides before a forecast rainfall event. Almost all of the fungicides used are protectants, which protects chickpea from Ascochyta blight infections for about 14–18 days when applied before a rainfall event (Pritchard, 2000). Protective fungicides are recommended as a main disease management strategy, and post infection fungicides have been found ineffective unless applied within 48 h of the rain (when paddocks are often too wet for ground rigs) (Moore et al., 2019). Australian chickpea farmers often have to start applying fungicides several days before a rain event because their paddocks are often larger than 1000 ha (a 24 m ground rig travelling at 20 kph will cover about 380 ha in an 8 h period). However, protective fungicide applications are often missed due to the unavailability of spray contractors, machinery breakdown, insufficient time to spray the crop prior to a rain event, and rain occurrence when it is not predicted (Moore et al., 2019). Little is known about the rainfall threshold for *A. rabiei* conidial release and dispersal, therefore crops often get sprayed when they do not need to be, resulting in unnecessary use of fungicides. The rainfall threshold is the minimum amount of rain required for conidia dispersal resulting in sufficient leaf wetness for germination and successful penetration of host tissues (Diggle et al., 2002). Rainfall threshold and disease intensity in turn are directly related to the inoculum density, i.e., less rain can cause more crop damage when inoculum density is high (Kaiser, 1992). Growers therefore need information on the likely interaction between rain duration and amount and the inoculum density that triggers fungicide spraying to make cost effective Ascochyta blight management decisions. At present, a 2 mm rain per day rainfall threshold value is used in an Ascochyta blight spread model used in Australias (Coventry, 2012). However, this value has not been determined experimentally. Information on the approximate rainfall threshold value from this study will inform Ascochyta blight models, resulting in more accurate disease prediction.

A previous study used large (10 × 2 m) chickpea plots with 100% plant infections to obtain an accurate estimate of the distance *A. rabiei* conidia travel in wind-driven rain (Khaliq et al., 2020). They found that greater inoculum density caused conidial dispersal to larger distances (0–75 m) from the inoculum source. Lesions were recorded on trap plants in rain amounts as low as 0.8 mm (Khaliq et al., 2020). To obtain more insight into a rainfall threshold, studies should use limited inoculum density and emphasis should be placed on capturing variable rain amount and duration.

Studies aimed at determining the relationship between rain and splash dispersal have largely been based on generating rain using a rain simulator. Rain simulators are useful for reproducing the timing and intensity of simulated rain, but simulated rain events do not necessarily represent the intrinsic characteristics (duration and frequency of occurrence) of natural rain events (Madden et al., 1996), as natural rain events are a combination of dry and wet spells, not a single wet spell. A rainfall event is defined as a sequence of rainy and rainless periods (He et al., 2020). Despite rain being the main driver of Ascochyta blight spread in Australia (where ascospores are absent), and despite being the sole determinant of making decisions related to fungicide application, the relationship between the intrinsic characteristics of natural rain and Ascochyta blight development has not been investigated. This research was conducted to investigate the relationship between natural rain intensity (mm/h) and Ascochyta blight development.

## Methods

### Experimental design and spore trapping

This study was conducted at the agriculture field site (Ag Plot) at the University of Southern Queensland, Toowoomba, from March to December 2020. The experiment was stopped between April 1 to July 1 due to COVID-19 restrictions, and then resumed again when restrictions were lifted. The relationship between rain intensity and Ascochyta blight development was determined by counting Ascochyta blight lesions on 21-day-old chickpea plants, placed around the inoculum source, during rainfall events of variable intensities. A rainfall event is hard to define, and its definition varies across disciplines. Definitions of a rainfall event across different disciplines have included: An event that produced point total precipitation of at least 500 mm over at most seven days (Nielsen-Gammon et al., 2005); a continuous rainfall without intermittence or at most 5 h pause (Yu et al., 2013); and a 5 day rainfall accumulation in excess of 1 mm (Brocca et al., 2014). For the purpose of this study, a rainfall event is defined as a period with no more than a 24 h break in between rain spells. A 24 h break between rain spells was chosen as a criterion because of the severe drought in the region for about three years proceeding this trial, so we were expecting less frequent rains. The largest gap between rain spells within a rainfall event was 21 hours (rainfall event 4), followed by 14 h (rainfall event 5) and 10 h (rainfall event 6). Commercial disease-free chickpea seed was used for growing chickpea plants. Infested residue pieces were sourced in the previous year from volunteer chickpea plants in a wheat paddock. 7 g of infested chickpea residue was placed at the soil surface in a 1 m^2^ plot and three pots (3 trap plants per pot) of a susceptible chickpea cultivar ‘Kyabra’ were randomly placed at each principal direction (north, south, east, west) of the 1 m^2^ plot (total 12 pots) preceding a forecasted rainfall event. A 1 m^2^ thin copper wire mesh was placed over the infested residue and pots were slightly set into the ground to prevent loss by wind. Trap plants were placed in all four directions of the infested stubble to account for the changing wind directions during rainfall events. The inoculum load on the infested residue pieces was not assessed precisely but efforts were made to select infested residue with approximately the same number of lesions through visual inspection. Emphasis was placed on capturing smaller rainfall events to get more insights into the approximate rainfall threshold. After exposure to a rainfall event, trap plants were transferred to a controlled temperature room set at 20 °C for 48 h (100% humidity). After a 48 h incubation period, trap plants were transferred to a glasshouse with temperature set at 20 °C to allow lesion development. Plants were watered daily with a hand sprinkler, and extreme care was taken to prevent splash dispersal to nearby plants. The number of Ascochyta blight lesions were counted on all trap plants after 14 days (± 2 days). The 1 m^2^ plot was cleaned thoroughly and the previously used infested residue were replaced with fresh residue (to account for inoculum depletion) for the next rainfall event. The process was repeated for a total of 7 rainfall events and the date and time trap plants were deployed and removed from the Ag Plot, and the date lesions were counted on trap plants were recorded (Table 1).

**Table 1:** Date and time trap plants deployed to and removed from agriculture field site (Ag Plot) of the University of Southern Queensland, Toowoomba, Queensland, with the date chickpea trap plants were assessed for lesions for each rainfall event. Lesions were counted after 14 days (± 2 days) after incubating plants

Rainfall event	Date deployed	Time deployed	Date removed	Time removed	Assessment date
1	24/02/2020	19:10	25/02/2020	15:30	19/03/2020
2	28/02/2020	17:00	01/03/2020	9:10	20/03/2020
3	03/03/2020	20:10	04/03/2020	16:50	22/03/2020
4	14/07/2020	13:00	29/07/2020	16:00	12/08/2020
5	06/08/2020	15:00	10/08/2020	10:00	25/08/2020
6	15/08/2020	7:10	17/08/2020	11:00	02/09/2020
7	24/11/2020	8:00	26/11/2020	16:00	10/12/2020

For the entire duration of the experiment, meteorological data were recorded by an automated weather station ‘WeatherMation’ (https://www.weathermation.net.au/WMLogin.aspx) located within 200 m of the experimental location. The data collected comprised air temperature (°C), rain (mm), relative humidity (%), wind speed (ms^−1^) and wind direction (°); all recorded at 10-minute intervals.

### Statistical analyses

The influence of total rain, rain duration (sum of durations of individual rain spells within a rainfall event, not the total duration trap plants were deployed in the Ag plot) and average wind speed on the total number of lesions recorded on trap plants per pot during each rainfall event was determined by fitting generalised linear mixed models using the `glmmTMB()` function of the ‘glmmTMB’ package version 1.0.2.9000 (Brooks et al., 2017) in the R programming language version 4.1.0 (R Core Team, 2021).

A quasipoisson family was used because preliminary analyses showed the data to be overdispersed (i.e., variance was greater than mean). The overdispersion was tested using Bolker’s custom function ‘overdisp_fun` (Bolker, 2021). The predictors total rain, rain duration and wind speed were included as fixed effects and the predictor rainfall event (total 7 rainfall events) was included as a random effect to compare directly the influence of total rain, rain duration and mean wind speed on the number of lesions recorded. The best fit model was selected using Akaike’s Information Criterion (AIC) (Akaike, 1974). Model diagnostics was performed using the DHARMa package version 0.4.1. The DHARMa package uses a simulation-based approach via the function `simulateResiduals` to create readily interpretable scaled residuals for glmms (Hartig, 2019).

Data were processed and visualised in the R programming language using ‘tidyverse’ version 1.3.1(Wickham et al., 2019), ‘lubridate’ version 1.7.10 (Grolemund & Wickham, 2011), ‘gridExtra’ version 2.3 (Auguie et al., 2017) and ‘clifro’ version 3.2.5 (Seers & Shears, 2015). Mean wind speed was calculated using a ‘circular.averaging’ function from ‘SDMTools’ version 1.1.221(VanDerWal et al., 2014).

## Results

**Fig. 1:**
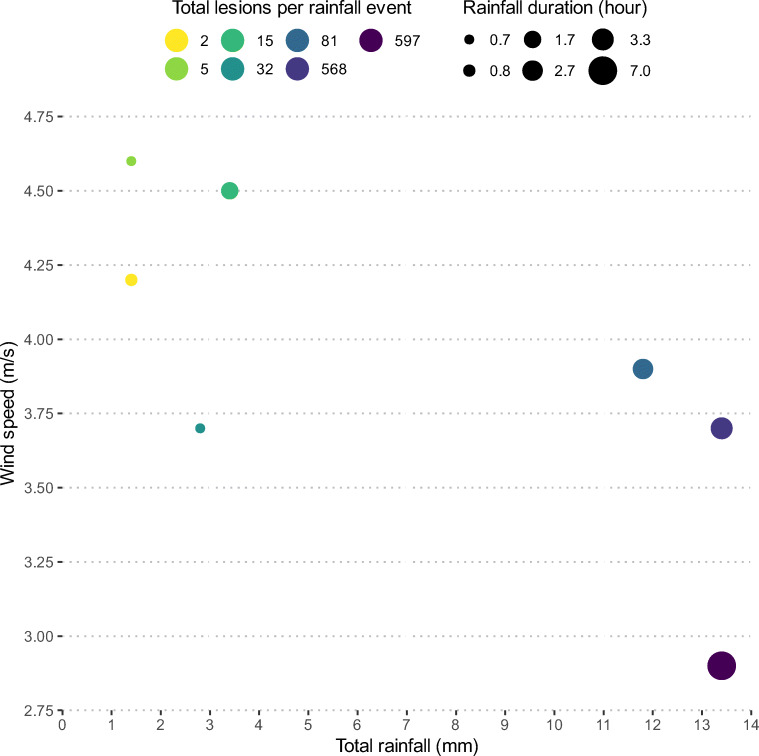
Total number of lesions per pot for 12 pots recorded for seven rainfall events as indicated by the point colour. Total rainfall (mm) is displayed on the x-axis with wind speed (m/s) on the y-axis. The point size relates to the duration of the rainfall event (hour). The number of lesions increased as the rainfall duration and amount both increased but decreased with increasing wind speed

**Fig. 2:**
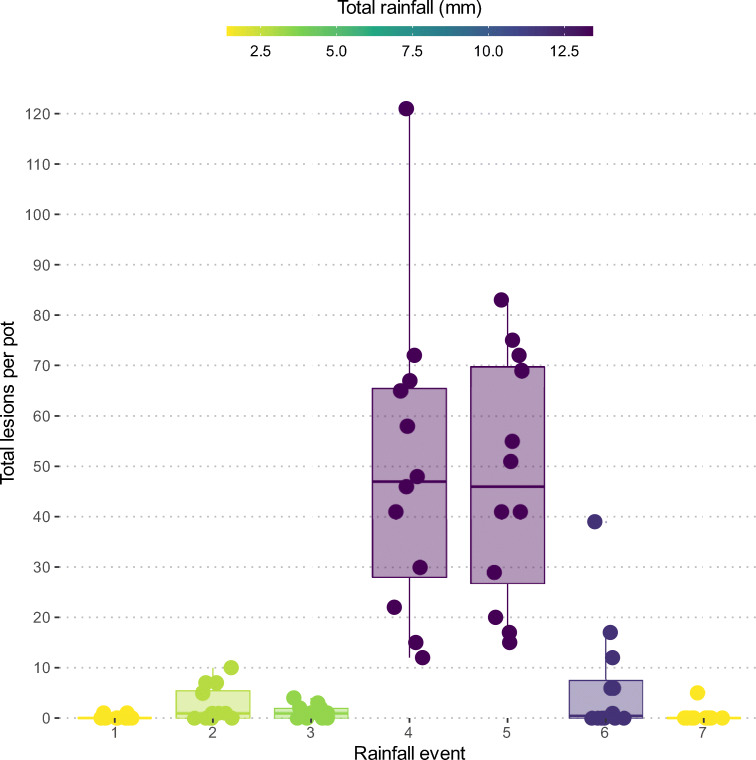
Box plots showing total number of lesions per pot for 12 pots recorded on chickpea trap plants during 7 different rainfall events. The highest number of lesions were recorded during rainfall event 4, 121 in one pot

The total number of lesions recorded on trap plants varied greatly among rainfall events (Table 2, Fig. 1). The number of lesions significantly (p < 0.05) increased with increasing rain amount. The highest number of lesions was recorded during rainfall event 4 (Fig. 2). Lesions were recorded in rain amounts as low as 1.4 mm and a rain duration as short as 0.7 h (Table 2). The number of lesions recorded tended to increase with an increasing rain duration, but the effect was non-significant (Fig. 1). For instance, more lesions (597) were recorded during rainfall event 4 than rainfall event 5 (568), although the same rain amount occurred during both rainfall events (Table 2). Conversely, the number of lesions recorded tended to decrease with increasing wind speed (Fig. 1), although the influence was statistically non-significant. This was particularly evident during rainfall events 2 and 3 where more lesions (32 vs. 15 lesions) were recorded during rainfall event 2, although higher rain amount (3.4 mm vs 2.8 mm) and duration (1.7 vs 0.7 h) was observed during rainfall event 3 (Table 2).

**Table 2:** Amount and duration of rain, mean relative humidity, mean wind speed, mean temperature and total lesions recorded on chickpea trap plants during each rainfall event

Rainfall event	Total rain (mm)	Rain duration (hours)	Mean relative humidity (%)	Mean wind speed (m/s)	Mean temperature (°C)	Total lesions
1	1.4	0.8	89.6	4.2	19.6	2
2	2.8	0.7	83.2	3.7	21	32
3	3.4	1.7	90	4.5	20.3	15
4	13.4	7.0	76.8	2.9	10.5	597
5	13.4	3.3	82.9	3.7	10.6	568
6	11.8	2.7	82.9	3.9	10.3	81
7	1.4	0.7	70.3	4.6	21.2	5

**Fig. 3:**
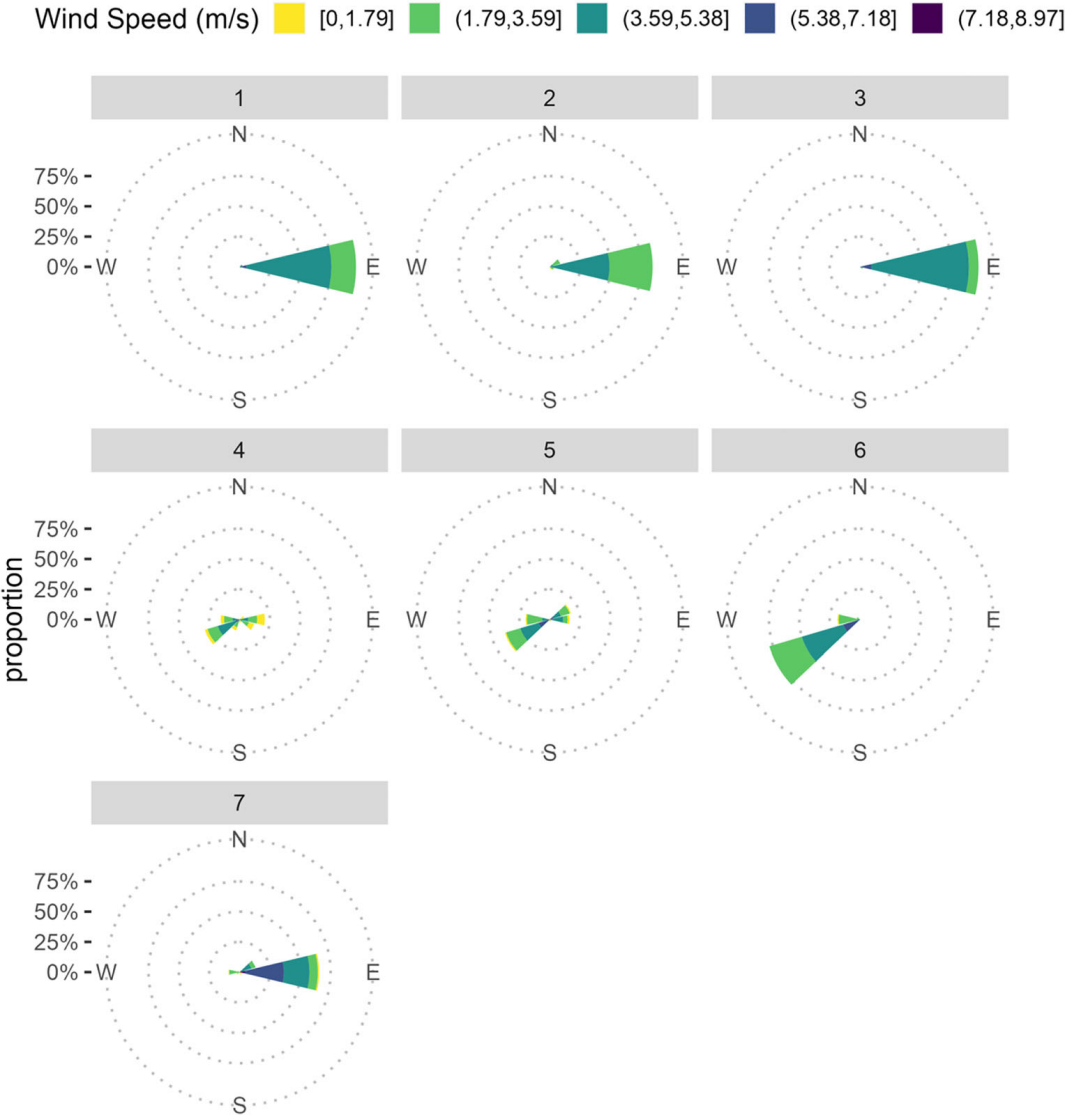
Wind roses showing daily average wind speed and direction during each rainfall event. The notation ‘[]’ represents a ‘closed interval’ indicating an interval is inclusive of both lower and upper values, whereas the notation ‘(]’ represent ‘half open interval’ indicating an interval is exclusive of the lower value but inclusive of the upper value

Mean wind speed, relative humidity and temperature varied during each rainfall event. Mean wind speed over the entire duration of the experiment ranged from 2.9 to 4.6 m/s, mean relative humidity ranged from 70.3 to 90% and mean temperature ranged from 10.3 to 21 °C (Table 2). Wind was not blowing in all directions during rainfall events. Wind was mostly blowing from the east during rainfall events 1, 2, 3, 7 and from the southwest during rainfall events 4, 5 and 6 (Fig. 3). DHARMa diagnostics showed that the data met the best fit model assumptions. The model fit for total rain, rain duration and wind speed is shown in Fig. 3.

## Discussion

To the best of our knowledge, this study is the first to investigate the effect of natural rain intensity and intrinsic attributes (wind direction and wind speed) associated with rain on Ascochyta blight in chickpea development, using limited inoculum density. The number of lesions recorded on trap plants increased with increased rain amount and duration, whereas the reverse was true for the relationship with wind speed. That is, the number of lesions recorded on trap plants decreased as wind speed increased.

The significantly greater number of lesions recorded on trap plants with increasing rain amounts is not surprising as increasing rain amount releases more conidia, which are then dispersed to neighbouring plants by rain splash (Kimber, 2002), or to longer distances (at least 75 m) by wind-driven rain (Khaliq et al., 2020). Rain not only releases conidia from pycnidia and disperses them, it also facilitates conidial germination, penetration and subsequent infection by keeping host tissues wet. *Ascochyta rabiei* requires a wetness period of at least 6–10 h to infect (Moore et al., 2016), and further disease development is very limited when relative humidity is less than 86% (Navas-Cortés et al., 1998). Lesions were recorded on trap plants in rain amounts as low as 1.4 mm. A previous study recorded lesions on trap plants in rain amount as low as 0.8 mm (higher inoculum density was used in the study, i.e., 10 × 2 m chickpea plots with 100% plant infections) (Khaliq et al., 2020). Conidial dispersal in low rain amounts suggests that conidial dispersal is more dependent on the occurrence of rain rather than the amount. A similar spore dispersal pattern has been observed for the sexual spores (ascospores) of *A. rabiei*, where ascospore discharge was more dependent on the occurrence of rain rather than the amount (Trapero-Casas et al., 1996). Of the different environmental factors affecting Ascochyta blight development, rain has been reported to have the major influence (Kimber et al., 2007).

The number of lesions recorded on trap plants tended to increase with an increasing rain duration. The positive effect of an increased rain duration on conidial dispersal was especially evident during rainfall event 4 where more lesions (597) were recorded than for rainfall event 5 (568), although the same rain amount (13.4 mm) occurred during both rainfall events. It is likely that longer duration rain provided sufficient kinetic energy for longer duration and dislodged more conidia without quickly depleting the inoculum supply. Consistent with the positive effect of an increasing rain amount, longer duration rainfall events can further increase lesion development by providing an adequate amount of wetness period to facilitate conidial germination, penetration into host tissues and subsequent infection. Although there is no information in relation to Ascochyta blight in chickpea, the positive influence of an increasing rain duration on spore dispersal has been well established for other plant pathogens (Ntahimpera et al., 1997; Sache, 2000). Compared to quick thunderstorms, low intensity and intermittent rains provide the most conducive conditions for spore dispersal without depleting the inoculum supply (Sache, 2000). The positive influence of an increasing rain duration on the number of lesions recorded on trap plants was statistically non-significant in the current study, which can be attributed to a small sample size.

Splash dispersal is linked with other environmental factors associated with rain, such as wind speed and wind direction (Khaliq et al., 2020). Increasing wind speed had a statistically non-significant negative influence on the number of lesions recorded on trap plants in the current study. The non-significant effect of wind speed on the number of lesions recorded on chickpea trap plants, distributed at the distances of 0, 10, 25, 50 and 75 m from infected chickpea plots, has been established previously (Khaliq et al., 2020). Wind speed also had no significant effect on the spatial and temporal Ascochyta blight progress from primary infection foci (i.e., four infested stubble pieces) placed at the center of a 20 m × 20 m plot (Khaliq, 2021). The non-significant effect of wind speed on both conidial and disease spread can be attributed to the absence of the sexual spores of *A. rabiei* in Australia. Ascospores are primarily dispersed up to hundreds of metres to kilometres by wind (Kaiser & Küsmenoglu, 1997; Trapero-Casas & Kaiser, 1992), while conidia are mainly splash dispersed to neighboring plants (Khan, 1999). However, the negative effect of increasing wind speed on the number of lesions recorded on trap plants in the current study is unlikely to reflect spore dispersal dynamics under field conditions, as a 1 m^2^ plot was used in the current study. It is likely that conidia did not fall on trap plants and were dispersed up and through the air as the wind speed tended to increase. Farmers` paddocks are usually large (> 100 ha), so disease outbreaks are possible when rain is accompanied by wind. For splash dispersed pathogens, the effect of wind and rain on spore dispersal is not mutually exclusive. That is, rain provides the mechanism to remove spores and splash disperse them shorter distances, while wind can transfer spores upward and across fields (Khan, 1999). It was hypothesized that if aerosols enter the turbulent boundary air, then wind can disperse conidia to distances that are comparable to ascospores (Coventry, 2012). The question whether conidia would remain viable after being transported to longer distances by turbulent wind remains unanswered, although Armstrong-Cho et al. (2004) found that *A. rabiei* conidia could withstand intermittent dry periods for up to 24 h after inoculation in controlled environmental conditions.

**Fig. 4:**
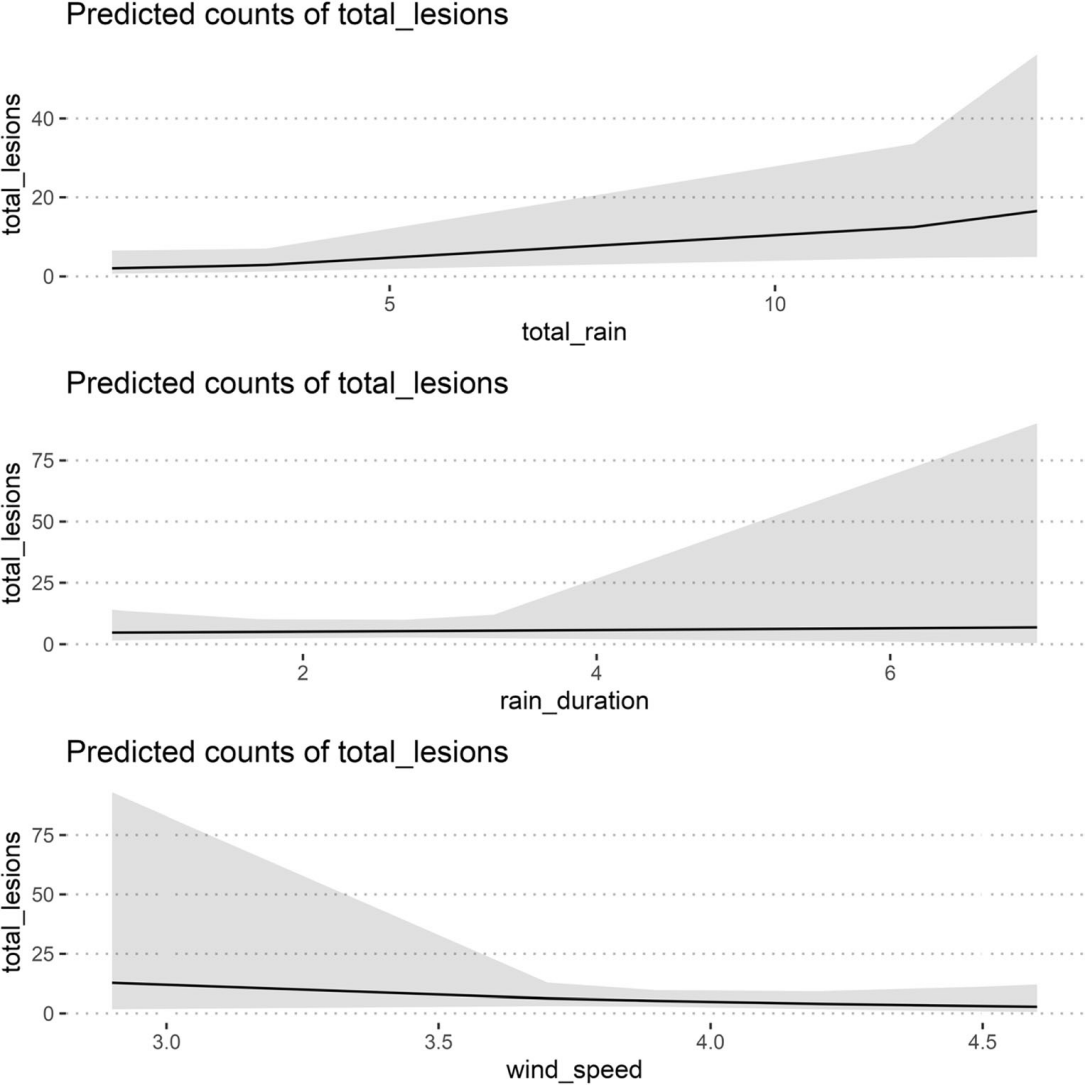
Generalised linear mixed models showing the influence of total rain, rain duration and average wind speed on the number of lesions recorded on chickpea trap plants

Wind was not omni-directional and was mostly blowing from the east during rainfall events 1, 2, 3, 7 and from the southwest during rainfall events 4, 5 and 6 (Fig. 4). The influence of wind direction was not directly investigated in the current study as the model failed to converge when a wind direction predictor was included in the model. However, the influence of wind direction on Ascochyta blight in chickpea has been well established. Wind direction is responsible for the directional spread of the disease when conidia in aerosols are blown in a particular direction (Coventry, 2012). Studies conducted on the spatial and temporal disease progress from primary infection foci showed that disease was more severe in the downwind direction of the primary infection foci (Coventry, 2012; Kimber et al., 2007). The tendency of wind to blow in all directions resulted in a uniform disease spread across plots (Coventry, 2012; Khaliq, 2021).

Our study shows that small rain amounts, shorter duration rains or a limited amount of primary inoculum is not a barrier in conidial dispersal and host infection. It is difficult to determine an exact rainfall threshold for spraying fungicides, as rainfall threshold can vary with rain intensity, the time of the day, inoculum density and the level of resistance of the cultivar. However, conidial dispersal in rain amounts as low as 1.4 mm (using limited inoculum density) suggest that the current 2 mm rainfall per day rainfall threshold used in Ascochyta blight models is not accurate for susceptible cultivars, especially when inoculum density is higher. Previous epidemiological studies have shown that limited inoculum is not a constraint in the development of an epiphytotic disease under conducive conditions (Coventry, 2012; Kaiser, 1992; Khaliq, 2021; Kimber et al., 2007). It has been shown that less than 1% of primary infection can have devastating effects on susceptible cultivars under cool and wet conditions (Kaiser, 1992); and single infection foci (four infested stem pieces placed at the center of the plots) were enough to spread disease across whole plots in a rainy season (Coventry, 2012; Khaliq, 2021). Successful disease management in chickpea should involve an integrated disease management approach, including paddock hygiene, maintaining 1–4 years rotation, sowing the best available resistant cultivars, seed treatment and strategic use of fungicides (Lyon, 2017). Our recommended strategic use of fungicides involves spraying before the occurrence of the first rain event after crop emergence, three weeks after crop emergence, or at the three branch crop growth stage, whichever occurs first. In low-risk areas, the crop should be monitored throughout the growing season, especially 10–14 days after a rain event, and fungicides should be applied if Ascochyta blight is detected. In high-risk (high rainfall) areas, it is recommended to spray fungicides every 14–21 days throughout the growing season (Pritchard, 2000).

It should be noted that the main aim of this study was to determine the interaction between natural rain amount, duration and limited inoculum density that triggers conidial dispersal and host infection to inform farmers and Ascochyta blight prediction models (rainfall threshold value for predictive models). The effect of environmental factors (wind direction and wind speed) have been cursorily considered in this study because their influence cannot be completely ignored when studying splash dispersal under natural conditions. As described above, the effect of wind speed on the distance (0, 10, 25, 50 and 75 m) *A. rabiei* conidia travel from the source of infection has been described elsewhere (Khaliq et al., 2020). Likewise, the effect of environmental factors (wind speed, wind direction, relative humidity and temperature) on Ascochyta blight development from the date of inoculation until harvest, under field conditions, has also been determined previously (Khaliq, 2021). Also, trap plants were incubated at 90% humidity for 48 h and then transferred to a controlled temperature room (20 °C), therefore considering the effect of temperature and relative humidity was not required.

Further experiments with continuous or uninterrupted rain events at different times of day and/or night will give more insights into the effect of rain intensity and time of the day/night on the disease development, although such experiments will be difficult to conduct with natural rain. Leaf wetness sensors may be used to measure the duration of leaf wetness produced by different amount of continuous or uninterrupted rain. Since rainfall thresholds for spore dispersal can vary with the level of resistance of chickpea cultivars, a larger scale study including cultivars of variable resistance may further increase our understanding on the relationship between natural rain intensity and Ascochyta blight development.
